# SARS-CoV-2 infection dysregulates the expression of clinically relevant drug metabolizing enzymes in Vero E6 cells and membrane transporters in human lung tissues

**DOI:** 10.3389/fphar.2023.1124693

**Published:** 2023-04-27

**Authors:** Chukwunonso K. Nwabufo, Md. Tozammel Hoque, Lily Yip, Maliha Khara, Samira Mubareka, Michael S. Pollanen, Reina Bendayan

**Affiliations:** ^1^ Department of Pharmaceutical Sciences, Leslie Dan Faculty of Pharmacy, University of Toronto, Toronto, ON, Canada; ^2^ OneDrug, Toronto, ON, Canada; ^3^ Sunnybrook Research Institute, Toronto, ON, Canada; ^4^ Ontario Forensic Pathology Service, Toronto, ON, Canada; ^5^ Department of Laboratory Medicine and Pathobiology, University of Toronto, Toronto, ON, Canada

**Keywords:** SARS- CoV-2, drug metabolism, drug transport, inflammatory response, Vero E6 cells, human lung tissues, drug metabolizing enzymes (DMEs), membrane transporters

## Abstract

SARS-CoV-2-mediated interactions with drug metabolizing enzymes and membrane transporters (DMETs) in different tissues, especially lung, the main affected organ may limit the clinical efficacy and safety profile of promising COVID-19 drugs. Herein, we investigated whether SARS-CoV-2 infection could dysregulate the expression of 25 clinically relevant DMETs in Vero E6 cells and postmortem lung tissues from COVID-19 patients. Also, we assessed the role of 2 inflammatory and 4 regulatory proteins in modulating the dysregulation of DMETs in human lung tissues. We showed for the first time that SARS-CoV-2 infection dysregulates CYP3A4 and UGT1A1 at the mRNA level, as well as P-gp and MRP1 at the protein level, in Vero E6 cells and postmortem human lung tissues, respectively. We observed that at the cellular level, DMETs could potentially be dysregulated by SARS-CoV-2-associated inflammatory response and lung injury. We uncovered the pulmonary cellular localization of CYP1A2, CYP2C8, CYP2C9, and CYP2D6, as well as ENT1 and ENT2 in human lung tissues, and observed that the presence of inflammatory cells is the major driving force for the discrepancy in the localization of DMETs between COVID-19 and control human lung tissues. Because alveolar epithelial cells and lymphocytes are both sites of SARS-CoV-2 infection and localization of DMETs, we recommend further investigation of the pulmonary pharmacokinetic profile of current COVID-19 drug dosing regimen to improve clinical outcomes.

## Introduction

Coronavirus disease 2019 (COVID-19) has killed over 6 million people and affected more than 600 million people worldwide, making it one of the deadliest pandemics of the 21st century (World Health organization, 2022). Although several vaccines are now available to protect against severe acute respiratory syndrome coronavirus 2 (SARS-CoV-2), full protection is not guaranteed for multiple reasons including evasion of neutralizing antibody by some variants of SARS-CoV-2 (Garcia-Beltran et al., 2021; Hoffmann et al., 2021; Madhi et al., 2021), breakthrough infection (Su et al., 2016; Krammer, 2020), and delay in reaching herd immunity (Our World in Data, 2021). Therefore, there is an urgent need to develop effective antiviral drugs that will complement the existing vaccines in improving the morbidity and mortality associated with COVID-19.

Currently, there is no cure for COVID-19; however, drugs such as remdesivir, molnupiravir, and nirmatrelvir have shown great promise relative to other candidates (Cascella et al., 2022). A major determining factor for the clinical efficacy and safety of these drugs is their ability to sufficiently distribute within the host and reach optimal therapeutic concentrations at disease target sites, particularly the lung tissue, the main organ affected by SARS-CoV-2 infection (Nwabufo and Aigbogun, 2022). For example, a previous study in rats found that the plasma levels of lopinavir were greater than its lung concentrations (Kumar et al., 2004), indicating that the drug may not be reaching the optimal pulmonary concentrations required to effectively eradicate SARS-CoV-2 virus. Yet, studies investigating the spatial distribution of COVID-19 drugs at the primary target cells (Type II alveolar epithelial cells) of SARS-CoV-2 infection within the lung tissues are still lacking.

The lung is heterogeneous, comprising about 40 different cell types with an unequal distribution of drug metabolizing enzymes and membrane transporters (DMETs), which have a lower expression and activity compared to their hepatic counterpart (Enlo-Scott et al., 2021a; 2021b). Alone or in synergy, these DMETs can alter the pulmonary concentration of drugs, rate and extent of their spatial distribution to disease target cells, accumulation in specific cell types and overall pulmonary drug retention, as well as absorption into the systemic circulation resulting in distinct local and systemic pharmacokinetic (PK)/pharmacodynamic (PD) profiles (Gustavsson et al., 2016; Ehrhardt et al., 2017).

Early response pro-inflammatory cytokines such as tumor necrosis factor (TNF)-α, interleukin-6 (IL-6), and IL-1β, are overproduced in a typical hospitalized COVID-19 patient and may be responsible for the acute respiratory distress syndrome, lung injury, and multiple-organ damage seen in some COVID-19 patients at severe stages of the disease (Aziz et al., 2020; Pilla Reddy et al., 2021; Rendeiro et al., 2021; Frisoni et al., 2022). Interestingly, IL-6 and IL-1β have both been implicated in the dysregulation (altering the normal levels) of DMETs (Dunvald et al., 2022), and their presence in COVID-19 pathophysiology warrants a similar investigation. Recent clinical studies have reported altered PK profiles for drugs such as midazolam (Le Carpentier et al., 2022), tacrolimus (Salerno et al., 2021), and lopinavir (Gregoire et al., 2020) in COVID-19 patients. These studies suggests that SARS-CoV-2-associated inflammatory response may be responsible for the altered PK profile. However, the molecular mechanism underlying SARS-CoV-2-mediated alteration of drug PK profile is yet to be demonstrated.

At the molecular level, proinflammatory cytokines may dysregulate the expression of DMETs through xenosensing regulatory proteins including pregnane X receptor (PXR), constitutive androstane receptor (CAR), nuclear factor kappa B (NF-kβ), phosphorylated signal transducer and activator of transcription 3 (pSTAT3) that control transcription (Wu and Lin, 2019; Stanke-Labesque et al., 2020; Dunvald et al., 2022). Also, inflammation-mediated damage of pulmonary cells housing DMETs, as well as recruitment of pulmonary immune cells such as macrophages (which also house DMETs) in response to SARS-CoV-2 infection could also alter local drug PK/PD profile (Nwabufo and Bendayan, 2022). In general, all these effects associated with an immune response to SARS-CoV-2 infection could lead to distinct PK/PD profiles in peripheral tissues and systemic circulation with potential clinical drug safety and efficacy issues for COVID-19 patients, especially those with polypharmacy.

**FIGURE 1:**
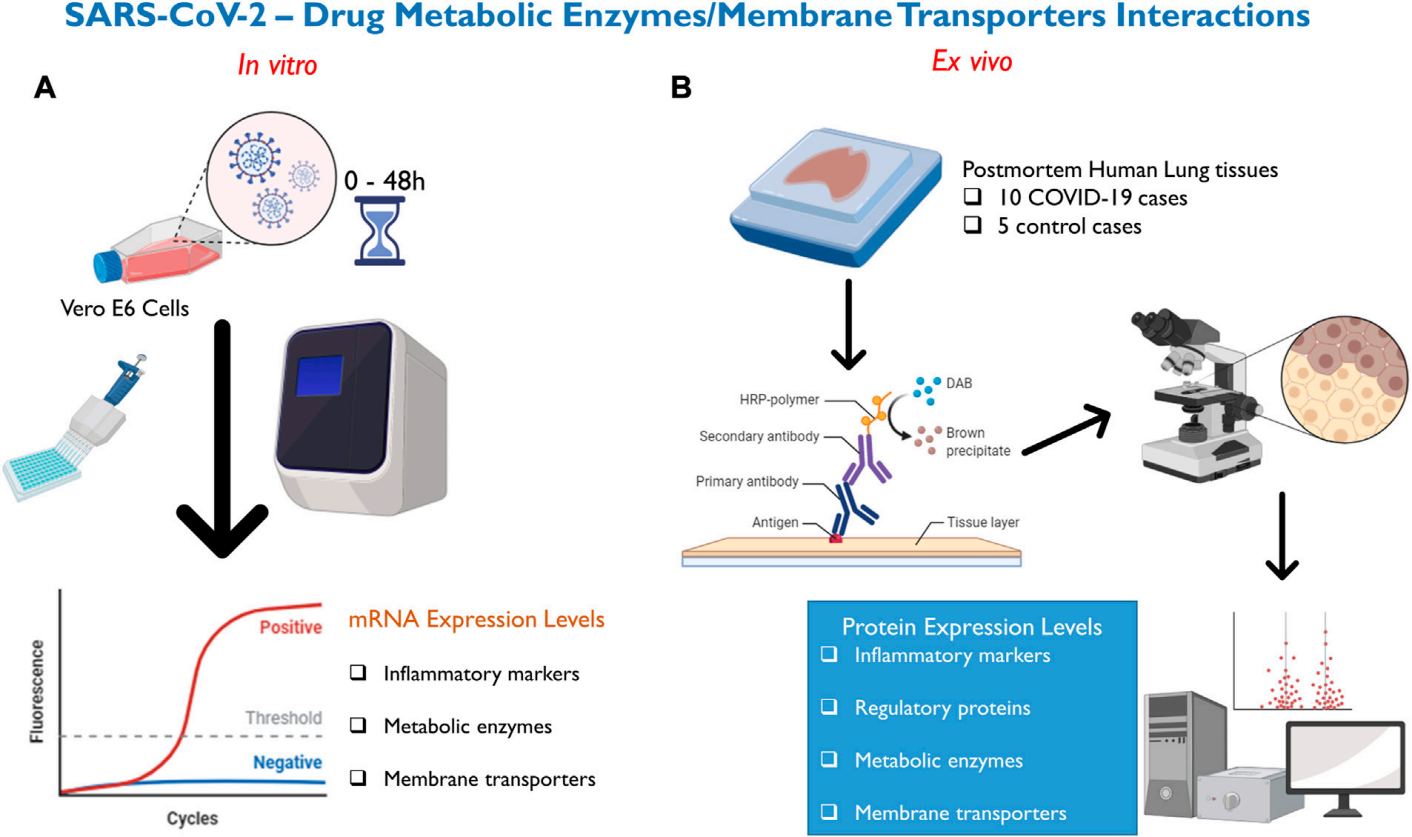
Methods deployed for investigation of SARS-CoV-2—DMETs interactions. **(A)** Vero E6 cells were infected with SARS-CoV-2 virus and cell pellets were collected at 0-, 6-, 24-, and 48- hours post-infection. Subsequently, relative mRNA expression of selected clinically relevant inflammatory markers (9), DMETs (12) was determined using qRT-PCR. **(B)** Chromogenic immunohistochemistry was used to localize and compare changes in protein expression of clinically relevant inflammatory markers (2), xenosensing regulatory proteins (4), DMETs (13) between COVID-19 and control postmortem human lung tissues.

In this present study (Figure 1), we investigated for the first time whether SARS-CoV-2 infection with D614G variant alters the mRNA expression of 9 inflammatory markers, 12 DMETs, in Vero E6 cells compared to mock. The D614G variant of SARS-CoV-2 was the most prevalent form of the virus at the onset of this study and is characterized by a mutation at position 614 in the spike protein which causes a change in amino acid sequence from aspartic acid to glycine (Korber et al., 2020). Several studies have shown that the D614G variant is more infectious than the original strain of the virus with increased spike protein binding to human cells (Korber et al., 2020; Yurkovetskiy et al., 2020; Zhang et al., 2020; Plante et al., 2021). Interestingly, the D614G mutation have been found in several variants of concern (VOI) including alpha (B.1.1.7), beta (B.1.351), delta (B.1.617.2), gamma (P.1), and omicron (BA.1, BA.2, BA.3, BA.4, BA.5) (Ou et al., 2022). Vero E6 cell line is derived from kidney epithelial cells of African green monkey and is one of the most used cell lines for studying SARS-CoV-2 virus because they express high levels of angiotensin-converting enzyme 2 receptor which is essential for cellular entry of the virus (Hoffmann et al., 2020; Rosa et al., 2021). This makes Vero E6 cell a good *in vitro* model for our study. More so, we have not identified any previous study that has examined the full panel of the inflammatory signature associated with SARS-CoV-2 infection or SARS-CoV-2-DMETs interactions in Vero E6 cells. Because the lung is the main organ affected by SARS-CoV-2 infection and may be prone to SARS-CoV-2-mediated dysregulation of DMETs, we further conducted a novel investigation of the cellular localization and changes in protein expression of 2 SARS-CoV-2-associated inflammatory markers, 4 regulatory proteins, and 13 DMETs in postmortem human lung tissues obtained from 10 COVID-19 patients compared to 5 age/sex-matched non-infected controls. We anticipate that any significant dysregulation will adversely affect the concentration of promising COVID-19 drugs in both peripheral tissues and systemic circulation, and may underpin the limited clinical efficacy and safety observed for several COVID-19 drug repurposing programs.

## Methods

### *In Vitro* SARS-CoV-2—DMETs interactions

#### SARS-CoV-2 infection in Vero E6 cells and RNA extraction

Vero E6 cells were kindly provided by Dr. Mubareka, Sunnybrook Research Institute, and the SARS-CoV-2 infection was performed in their laboratory. Vero E6 cells were seeded in T75 cm^2^ flasks to achieve 95% confluency the next day. Using a multiplicity of infection of 0.001 to avoid over-infection and excessive cell toxicity, cells were inoculated with 1.5 mL of SARS-CoV-2 virus containing the spike-protein D614G mutation. Mock controls were inoculated with DMEM media only. Cells were incubated at 37°C, 5% CO_2_ for 45 min and rocked every 10 min before inoculum was removed and topped up with 15 mL DMEM (Wisent #319-005-CL) containing 2% heat-inactivated FBS (Wisent #080450), 100IU penicillin- 100 μg/ml streptomycin and 2 mM L-glutamine (Wisent # 450-202-EL). Cell pellets were collected at 0-, 6-, 24-, and 48- hours’ post-infection (hpi). Cell pellets were prepared by removing the supernatant, washing with 10 mL cold PBS, and scraping the monolayer with 5 mL fresh PBS. Lifted cells were collected into a tube, and another 5 mL of fresh PBS was added to collect any remaining cells. Cells were spun at 1,000 g for 5 min at 4°C and kept in the −80°C freezer. RNA extraction was performed using the Qiashredder and RNeasy Mini Plus kit (Qiagen). RNA concentrations at an absorbance of 260 nm and purity at an absorbance ratio of 260/280 were quantified using Nanodrop One Spectrophotometer (Thermo Scientific).

#### Virus stock

The virus (S357_P2_LY) propagated in Vero E6 cells and contains spike-protein D614G mutation. Sequencing revealed that the virus stock has 2 deletions (7% in position 23,598% and 10% in position 23,628) in the polybasic furin cleavage site located on the S gene. Supplementary Table S1 shows a list of other single nucleotide variants and their frequencies.

#### Real-time quantitative polymerase chain reaction analysis

Real-time quantitative polymerase chain reaction (qRT-PCR) was used to measure the mRNA expression of selected inflammatory markers, and DMETs (Supplementary Table S2). 2 μg isolated RNA was treated with DNase I to remove residual DNA and then reverse transcribed to cDNA using a high-capacity reverse transcription cDNA kit (Applied Biosystems) according to the manufacturer’s instructions. Specific monkey TaqMan primers for selected inflammatory markers and DMETs (Supplementary Table S3) obtained from Life Technologies were used with TaqMan quantitative polymerase chain reaction biochemistry. All assays were performed in triplicates with *PPIB* (Peptidylprolyl isomerase B) and *GAPDH* (Glyceraldehyde-3-phosphate dehydrogenase; used for *CRP* and *IL10*) housekeeping genes as an internal control. For each gene, the critical threshold cycle (CT) was normalized to the housekeeping gene using the comparative CT method. Next, the difference in CT values (ΔCT) between the gene of interest and the housekeeping gene was then normalized to the corresponding ΔCT of the vehicle control (ΔΔCT) and the relative difference in mRNA expression for each gene was represented as 2^−ΔΔCT^.

### SARS-CoV-2—DMETs interactions in human lung tissues

The studies involving human participants were reviewed and approved by the office of the Chief Coroner at Ontario Forensic Pathology Service (Chief Forensic Pathologist, and Chief Coroner). Written informed consent for participation was not required for this study in accordance with the national legislation and the institutional requirements.

#### Autopsies and postmortem lung tissue processing

Autopsies were performed as per guidelines provided by the Ontario Forensic Pathology Service with appropriate infection isolation procedures. Histological samples obtained during the autopsy were fixed in 10% neutral buffered formalin for at least 24 h before processing.

#### Immunohistochemical analysis

Chromogenic immunohistochemistry (IHC) was used to localize and quantify the expression of SARS-CoV-2 virus, inflammatory markers, regulatory proteins, and DMETs (Supplementary Table S4) in postmortem human lung tissues obtained from 10 infected COVID-19 patients and 5 age/sex-matched non-infected controls (Table 1). IHC was performed on 4 μm formalin-fixed paraffin-embedded sections of lung tissues. Primary antibodies for the selected biomarkers (Supplementary Table S5) were detected using a secondary antibody and horseradish peroxidase-conjugated streptavidin (MACH 4 universal HRP kit, Biocare Medical, CA, United States), followed by color development with 3,3′-Diaminobenzidine (DAB; DAKO Cat# K3468). Subsequently, cell morphology and nuclei were visualized by counterstaining with hematoxylin and eosin (H&E). Reagent negative control (Supplementary Figure S1) and positive control (Supplementary Figure S2) were performed to determine specificity. All immunostained slides were imaged with an Aperio AT2 brightfield scanner (Leica Biosystems) at ×20 magnification on a standard slide dimension (1”×3″).

**TABLE 1:** Clinical and demographic characteristics for patients included in the immunohistochemistry study.

Sample ID	COVID-19 Status	Age	Gender	Cause of death
1	COVID-19	76	M	COVID-19
2	COVID-19	60	M	COVID-19
3	COVID-19	76	F	COVID-19; Chronic obstructive lung disease; Hypertensive cardiovascular disease
4	COVID-19	48	M	COVID-19
5	COVID-19	53	F	Complications of COVID-19 pneumonia (with saddle pulmonary embolism and deep vein thrombosis
6	COVID-19	37	M	COVID-19 with acute pulmonary thromboembolism
7	COVID-19	83	M	COVID-19; Atherosclerotic and hypertensive heart disease; Pulmonary emphysema
8	COVID-19	66	M	COVID-19; Diabetes mellitus; Essential hypertension
9	COVID-19	56	F	COVID-19
10	COVID-19	32	M	COVID-19
11	Non-COVID-19	32	M	Gammahydroxybutyrate toxicity
12	Non-COVID-19	41	M	Multiple drug toxicity (fentanyl, ethanol, and methamphetamine)
13	Non-COVID-19	59	F	Acute coronary thrombosis; intraplaque hemorrhage and rupture; atherosclerotic coronary artery disease
14	Non-COVID-19	66	M	Atherosclerotic heart disease
15	Non-COVID-19	74	M	Blunt impact head trauma

#### Absolute protein quantitation

HALO software v3.4 (Indica Labs) was used to analyze the entire lung tissue section of each slide across all the investigated biomarkers. The multiplex IHC (v3.1.4) algorithm was used to quantify percentage of DAB positive cells which comprised the total number of DAB positive cells relative to the total number of cells quantified in the images.

### Data analysis

All statistical analyses were performed using GraphPad Prism^®^ (version 8.0 for Microsoft Windows, Graph Pad Software, San Diego, CA, United States) with a significant difference defined as a *p*-value of 0.05 or less. All results for mRNA expression and protein quantification were expressed as mean ± standard deviation (SD) and mean ± standard error of the mean (SEM), respectively. An unpaired *t*-test was used to determine significant differences in mRNA expression between the SARS-CoV-2-infected and mock Vero E6 cells. The non-parametric two-tailed Mann-Whitney test was used to assess the differences in protein expression between COVID-19 and control postmortem human lung tissues for absolute quantitative IHC analysis.

## Results

### *In Vitro* SARS-CoV-2—DMETs interactions

#### SARS-CoV-2-associated inflammatory response in Vero E6 cells

**FIGURE 2:**
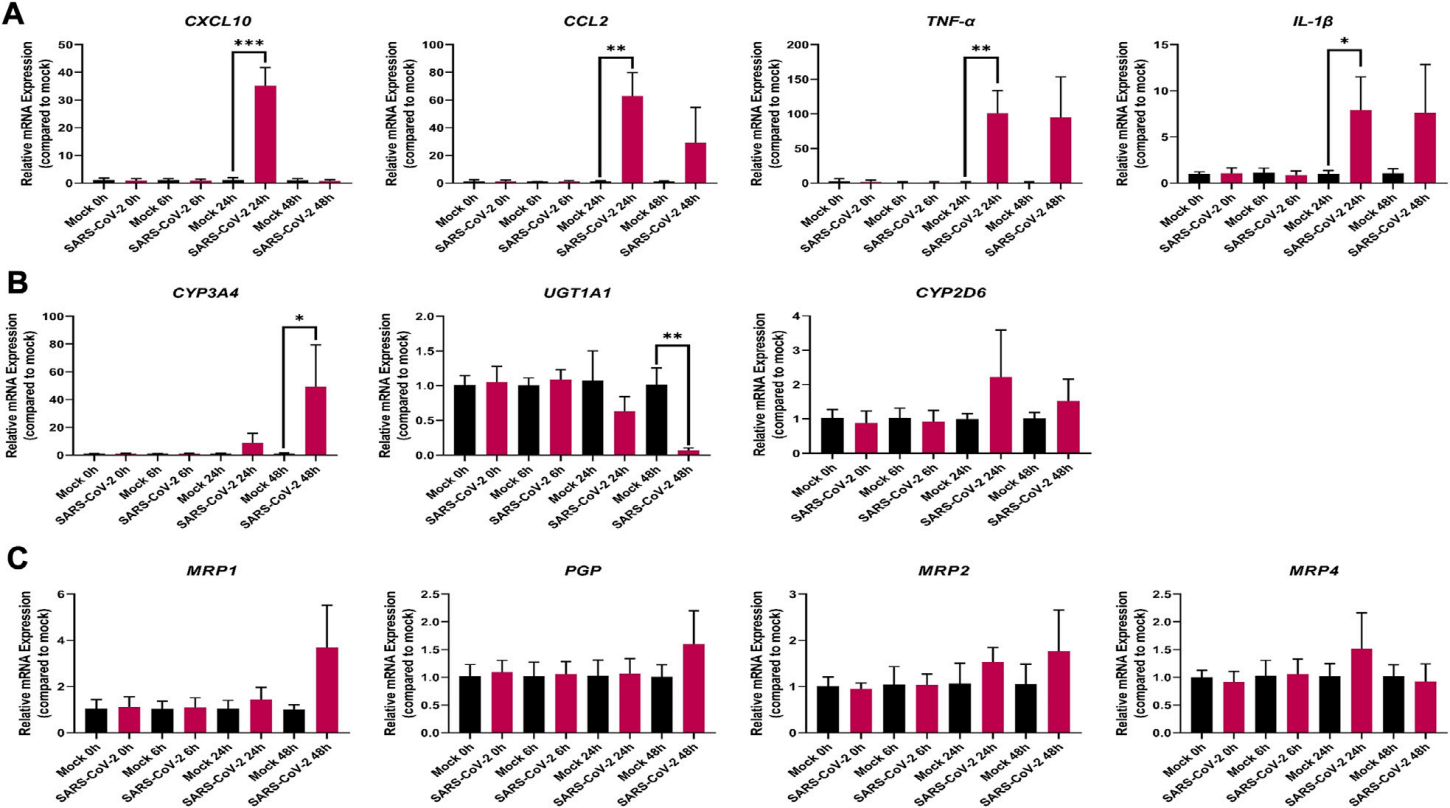
Effect of SARS-CoV-2 infection on the mRNA expression of selected **(A)** inflammatory markers; **(B)** drug metabolizing enzymes; and **(C)** membrane-associated drug transporters in Vero E6 cells. Relative mRNA expression was determined using qRT-PCR with normalization to the housekeeping gene and the mock. Results are expressed as mean ± SD from 3 independent experiments, and unpaired *t*-test was used to determine significant differences (*, *p* < 0.05; **, *p* < 0.01; ***, *p* < 0.001).

We observed significant changes in the mRNA expression level of inflammatory markers associated with COVID-19 severity including *IL-1β*, *TNF-α*, *CCL2*, and *CXCL10* in our SARS-CoV-2 infected Vero E6 cells (Figure 2A); suggesting that Vero E6 cells may be used to study the inflammatory events associated with COVID-19 and modelling the severe stage of the disease *in vitro*.

The mRNA expression of the pro-inflammatory cytokines, *IL-1β* and *TNF-α* was markedly upregulated by 7-(*p* < 0.05) and 81-(*p* < 0.01) fold, respectively, at 24 h (Figure 2A). Similarly, the mRNA expression of the chemokines, *CCL2* and *CXCL10*, was markedly increased by 56-(*p* < 0.01) and 29-(*p* < 0.001) fold, respectively, at 24 h in the infected SARS-CoV-2 Vero E6 cells (Figure 2A). However, at the 48-h mark, the mRNA expression level of *CXCL10* decreased to baseline in the infected SARS-CoV-2 Vero E6 cells (Figure 2A). No mRNA expression was observed for *CRP* and *IL-10* in both mock and infected Vero E6 cells (data not shown).

It is possible that species differences (humans and monkeys), as well as potential distinctions in the inflammatory events associated with the different variants of SARS-CoV-2 virus may be responsible for the lack of observable significant differences in the mRNA expression of *IL6, iNOS*, and *IFN- γ* (Supplementary Figure S3A) in the infected Vero E6 cells.

#### SARS-CoV-2—DMET interactions in Vero E6 cells

Out of the 7 investigated DMEs, only *CYP3A4* and *UGT1A*1 were significantly dysregulated at the mRNA level in SARS-CoV-2 infected Vero E6 cells (Figure 2B). At 48 h, the mRNA expression of *CYP3A4* was significantly upregulated by 50-fold (*p* < 0.05) in the infected Vero E6 cell (Figure 2B). At the same time point, *UGT1A1* mRNA expression was significantly downregulated by 0.5-fold (*p* < 0.001) in the infected Vero E6 cell (Figure 2B). No significant dysregulation of *CYP2D6* mRNA expression was observed (Figure 2B), and no mRNA expression was detected for *CYP1A2*, *CYP2B6*, *CYP2C8*, and *CYP2C9* in both mock and infected Vero E6 cells (data not shown).

None of the investigated membrane-associated drug transporters (*PGP*, *BCRP*, *MRP1*, *MRP2*, and *MRP4*) showed significant dysregulation in mRNA expression (Figure 2C; Supplementary Figure S3B).

### SARS-CoV-2—DMET interactions in human lung tissues

#### Clinical characteristics and demographic information

Postmortem human lung samples were obtained from 10 and 5 (age/sex-matched) COVID-19 and control patients, respectively (Table 1). COVID-19-related pathologies were the primary cause of death reported for the COVID-19 cases while no pulmonary pathologies were reported as the cause of death for the control cases. Comorbidities including pulmonary and cardiovascular diseases, as well as a metabolic disorder (Table 1) was observed in 3 of the COVID-19 cases. No additional information, for example, genetic polymorphisms of DMETs, cytokine panel, and intake of medications, were available for the COVID-19 and control groups.

#### COVID-19-related histopathological findings

**FIGURE 3:**
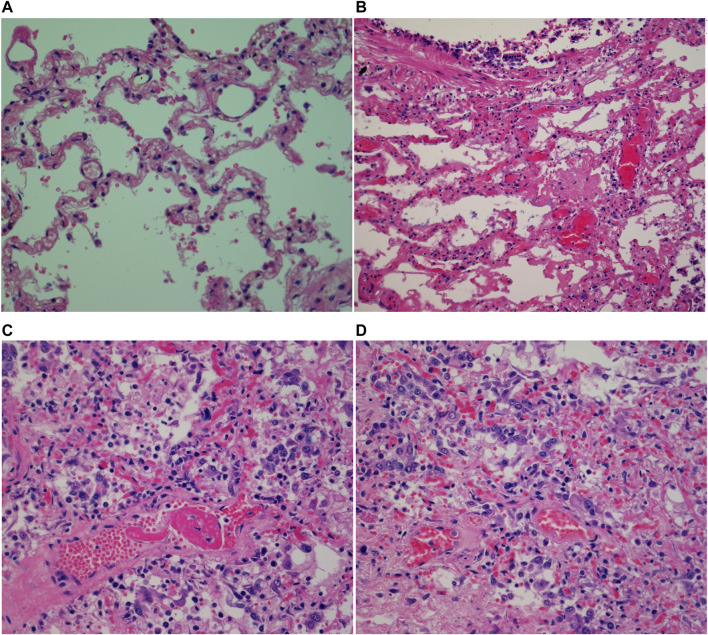
Micrographs of H&E stained postmortem human lung tissues (×40 magnification with an Olympus B×43 microscope) showing **(A)** normal human lung; **(B)** hyaline membrane formation; **(C)** perivascular lymphocytes; **(D)** COVID-19 organizing pneumonia.

Histological examination of the control cases did not reveal any significant pathologic abnormalities (Figure 3A). In COVID-19 cases, diffuse alveolar damage (DAD) was observed in two cases (Figure 3B). Assessment of preexisting lung disease was obscured by acute pathologies in some cases. For example, one COVID-19 case revealed multiple pathological changes: vascular hyperplasia, pigments in the alveolar spaces, thick alveolar septa, diffuse fibrosis, patchy areas of hyaline membranes, patchy type II pneumocyte hyperplasia, and focal areas of organizing COVID-19 pneumonia (Figures 3C, D). Another COVID-19 case had acute bronchitis, and 6 of the COVID-19 patients had hyaline membrane formation (Figure 3B). Pneumonia, ranging from acute to organizing phase (Figure 3C) was found in 7 COVID-19 cases. Five COVID-19 cases were also found to have inflammatory cells; one case had focal aggregation of chronic inflammatory cells, two cases had more acute and chronic inflammatory cells (Figure 3D), one case had mixed inflammatory infiltrates, and the fifth case had mixed inflammatory cells with more neutrophils. Two of the COVID-19 cases had unique features; one had more expanded air spaces and more diffuse intra-alveolar blood infiltration, while the other case had focal areas of consolidation (data not shown).

#### Localization of selected biomarkers in postmortem human lung tissues

**FIGURE 4:**
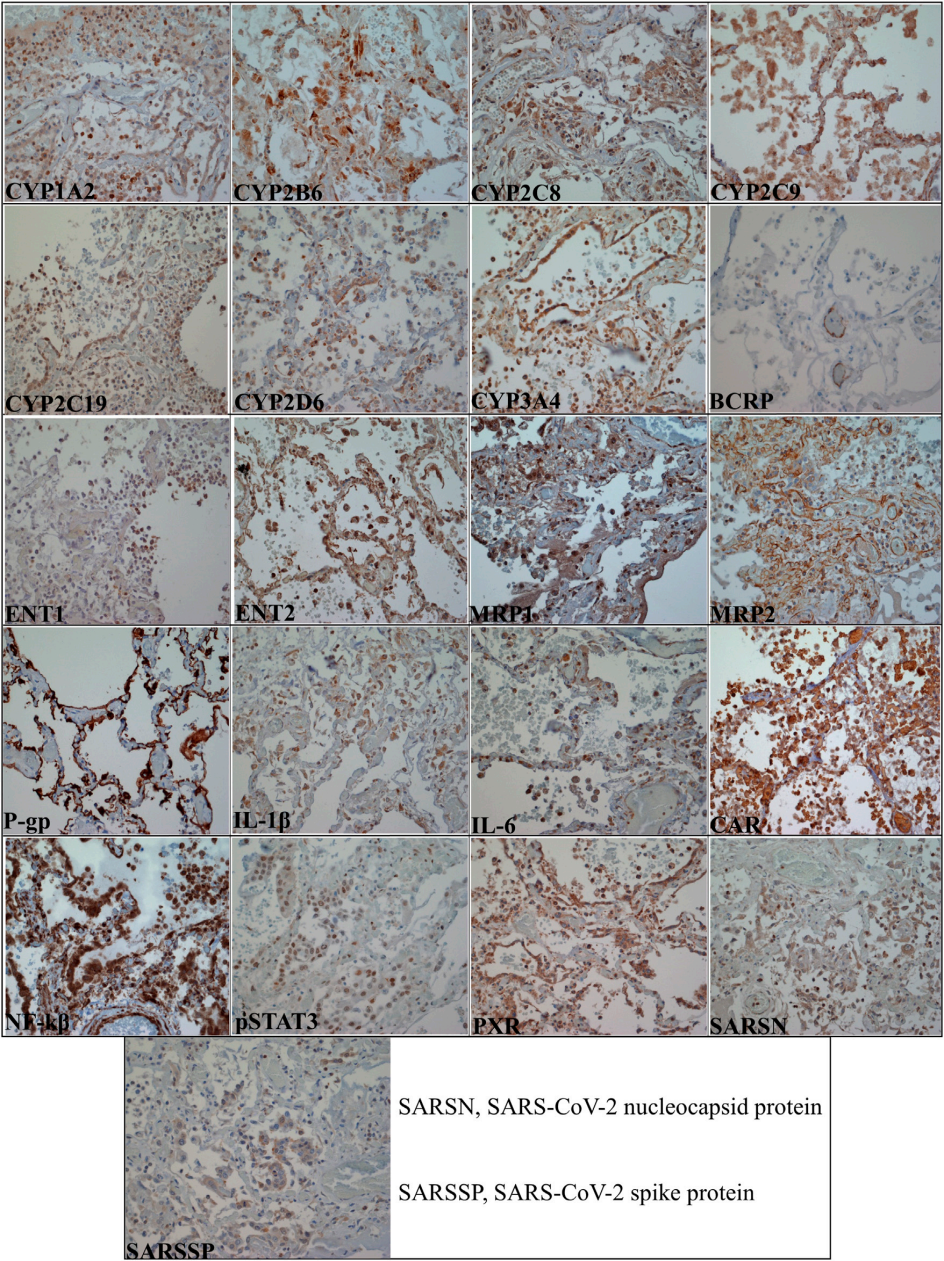
Micrographs of COVID-19 postmortem human lung tissue sections (×40 magnification with an Olympus B×43 microscope) showing positive staining in brown color for all the investigated biomarkers. Table 2 summarizes the pulmonary cellular localization of the investigated biomarkers.

Figure 4 shows the localization of the selected biomarkers (Supplementary Table S4) in COVID-19 postmortem human lung tissues while Table 2 summarizes the cellular expression of the biomarkers in each COVID-19 and control postmortem human lung tissues. Supplementary Figures S1, S2 are negative and positive control IHC images showing the good specificity of the antibodies used for the investigated biomarkers. In general, the investigated biomarkers are expressed in different pulmonary cell types with differences in cellular localization between COVID-19 and control cases (Figure 4; Table 2). The distinction in biomarker localization is driven by the infiltration of inflammatory cells such as lymphocytes and macrophages (Figure 4; Table 2). For example, CYP1A2 is predominantly expressed in the cytoplasm of alveolar epithelial cells for both COVID-19 (10/10) and control (5/5) lung tissues; however, it is distinctly expressed in the cytoplasm of intra-alveolar lymphocytes (9/10) and macrophages (5/10) of COVID-19 subjects (Figure 4; Table 2). Furthermore, there is a concordance in the pulmonary cellular localization of SARS-CoV-2 infection, inflammatory response, regulatory proteins, as well as DMETs. For instance, SARS-CoV-2 spike protein, nucleocapsid protein, IL-1β, IL-6, PXR, CAR, CYP2C9, CYP2C19, CYP2D6, and MRP1 are all more distinctly localized in the cytoplasm of lymphocytes in the COVID-19 but not control human lung tissues (Figure 4; Table 2), suggesting that SARS-CoV-2 infection may trigger an inflammation-mediated regulation of the expression of DMETs through regulatory proteins such as PXR and CAR in lymphocytes.

**TABLE 2:** Summary of the cellular localization of investigated biomarkers in postmortem human lung tissues.

Class of biomarkers	Biomarker	Cellular compartment	Pulmonary cells	COVID-19 cases	Control cases
*Drug metabolizing enzymes*	CYP1A2	Cytoplasm	Intra-alveolar lymphocytes	9/10	2/5
			Alveolar epithelial cells	10/10	5/5
			Macrophages	5/10	1/5
	CYP2B6	Cytoplasm	Alveolar epithelial cells	10/10	4/5
			Macrophages	3/10	3/5
			Lymphocytes	5/10	1/5
			Fibroblast	1/10	ND
		Nucleus	Lymphocytes	4/10	ND
	CYP2C8	Cytoplasm	Lymphocytes	6/10	3/5
			Macrophages	7/10	5/5
			Endothelial cells	8/10	3/5
			Fibroblast	3/10	ND
			Alveolar epithelial cells	8/10	4/5
			Bronchial epithelial cells	1/10	ND
	CYP2C9	Apical membrane	Alveolar epithelial cells	10/10	5/5
		Cytoplasm	Macrophages	9/10	3/5
			Fibroblast	1/10	ND
			Lymphocytes	3/10	ND
	CYP2C19	Cytoplasm	Alveolar epithelial cells	10/10	5/5
			Bronchial epithelial cells	3/10	4/5
			Lymphocytes	5/10	ND
			Macrophages	6/10	3/5
			Fibroblast	2/10	ND
	CYP2D6	Cytoplasm	Bronchial epithelial cells	3/10	3/5
			Lymphocytes	3/10	ND
			Macrophages	3/10	1/5
			Alveolar epithelial cells	8/10	4/5
		Cytoplasmic and circumferential membranous	Macrophages	ND	1/5
			Lymphocytes	1/10	ND
		Apical membrane	Bronchial epithelial cells	1/10	1/5
	CYP3A4	Cytoplasm	Lymphocytes	8/10	1/5
			Macrophages	2/10	3/5
			Alveolar epithelial cells	8/10	4/5
			Bronchial epithelial cells	3/10	1/5
*Membrane-associated drug transporters*	BCRP	Cytoplasm	Submucosal gland basement membrane	1/10	ND
			Macrophages	ND	1/5
			Endothelial cells	8/10	5/5
		Membranous	Macrophages	ND	2/5
	ENT1	Cytoplasm	Macrophages	1/10	2/5
			Subset of lymphocytes	6/10	2/5
		Circumferential membrane	Alveolar epithelial cells	ND	1/5
	ENT2	Apical membrane	Endothelial cells	2/10	ND
			Alveolar epithelial cells	4/10	3/5
		Circumferential membrane	Fibroblast	1/10	ND
		Cytoplasm	Macrophages	6/10	3/5
			Lymphocytes	7/10	1/5
			Alveolar epithelial cells	3/10	1/5
			Endothelial cells	1/10	ND
	MRP1	Cytoplasm	Lymphocytes	6/10	ND
			Macrophages	7/10	3/5
			Bronchial epithelial cells	2/10	1/5
			Alveolar epithelial cells	7/10	4/5
		Nucleus and cytoplasm	Intra-alveolar cells	1/10	ND
		Nucleus	Alveolar epithelial cells	9/10	5/5
	MRP2	Circumferential membrane	Bronchial epithelial cells	1/10	3/5
			Endothelial cells	10/10	4/5
			Macrophages	4/10	ND
			Lymphocytes	3/10	ND
			Alveolar epithelial cells	10/10	5/5
	P-gp	Cytoplasm	Endothelial cells	1/10	ND
		Circumferential membrane	Macrophages	2/10	ND
			Bronchial epithelial cells	4/10	5/5
			Alveolar epithelial cells	5/10	1/5
			Lymphocytes	1/10	4/5
			Submucosal gland epithelium	ND	2/5
		Apical membrane	Alveolar epithelial cells	8/10	4/5
*Inflammatory markers*	IL-1β	Cytoplasm	Lymphocytes	6/10	ND
			Alveolar epithelial cells	9/10	5/5
			Macrophages	5/10	4/5
			Endothelial cells	1/10	1/5
	IL-6	Cytoplasm	Neutrophils	1/10	ND
			Lymphocytes	9/10	ND
			Smooth muscles of blood vessels	2/10	ND
			Alveolar epithelial cells	8/10	4/5
			Bronchial epithelial cells	3/10	3/5
			Macrophages	3/10	3/5
			Scattered intra-vascular lymphocytes	ND	1/5
*Regulatory proteins*	CAR	Cytoplasm	Macrophages	7/10	4/5
			Lymphocytes	4/10	ND
			Fibroblast	2/10	ND
			Bronchial epithelial cells	ND	1/5
	NF-kβ	Cytoplasm	All the cells except the red blood cells	10/10	5/5
	pSTAT3	Nucleus	Alveolar epithelial cells	10/10	4/5
			Macrophages	4/10	ND
			Intra-alveolar lymphocytes	3/10	ND
			Lymphocytes	2/10	ND
			Endothelial cells	ND	1/5
		Cytoplasm	Macrophages	ND	2/5
	PXR	Cytoplasm	Smooth muscles of blood vessels	10/10	5/5
			Smooth muscles of bronchi	9/10	5/5
			Endothelial cells	10/10	4/5
			Bronchial epithelial cells	4/10	2/5
			Lymphocytes	8/10	ND
			Macrophages	2/10	3/5
*Viral proteins*	SARS-CoV-2 nucleocapsid protein	Cytoplasm	Lymphocytes	6/10	1/5
			Macrophages	2/10	3/5
			Alveolar epithelial cells	9/10	4/5
			Bronchial epithelial cells	ND	1/5
	SARS-CoV-2 spike protein	Cytoplasm	Lymphocytes	8/10	2/5
			Bronchial epithelial cells	2/10	4/5
			Alveolar epithelial cells	4/10	1/5
			Intra-vascular neutrophils	2/10	3/5
			Macrophages	1/10	3/5
			Intra-vascular lymphocytes	ND	1/5

#### Absolute quantitative analysis of protein expression

**FIGURE 5:**
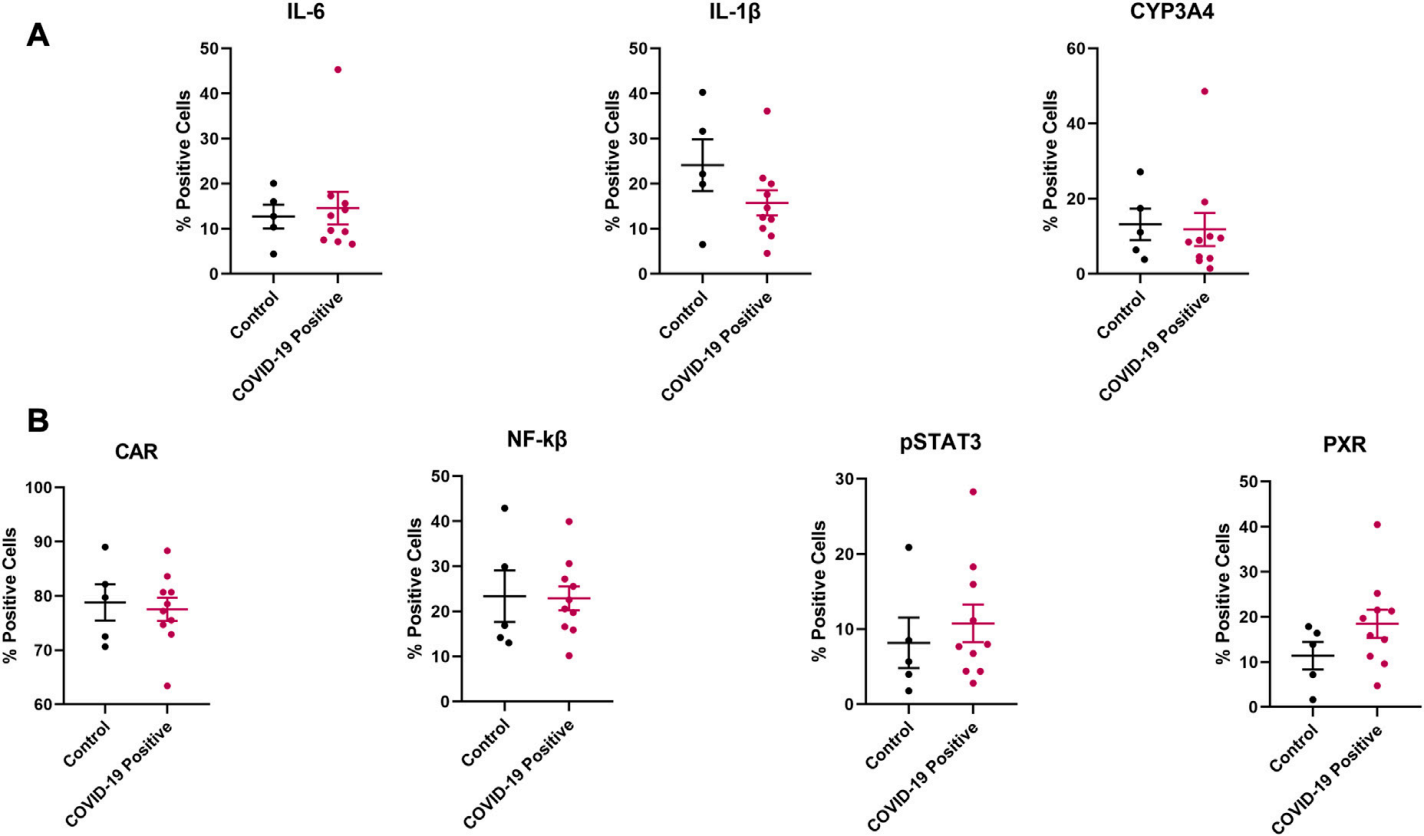
Effect of COVID-19 on the protein expression of selected **(A)** inflammatory markers and CYP3A4, **(B)** regulatory proteins in postmortem human lung tissues. Chromogenic images obtained from the immunostained tissue slides from each subject were analyzed using HALO software v3.4 (Indica Labs) for absolute quantitative IHC analysis. Results are expressed as mean ± SEM, and the non-parametric two-tailed Mann-Whitney test was used to determine the differences in protein expression between COVID-19 and control postmortem human lung tissues for absolute quantitative IHC analysis.

**FIGURE 6:**
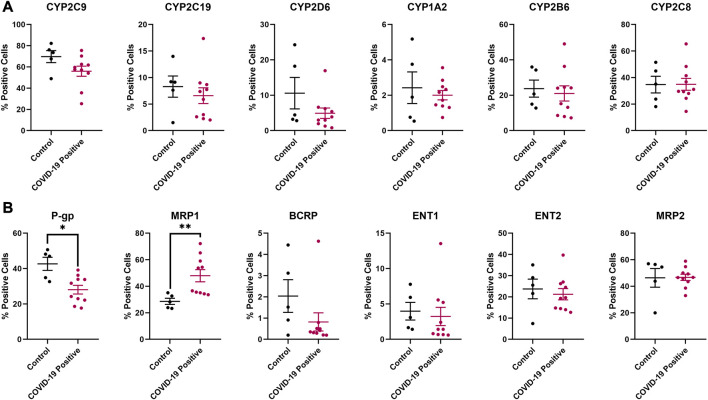
Effect of COVID-19 on the protein expression of selected **(A)** drug metabolizing enzymes and **(B)** membrane-associated drug transporters in postmortem human lung tissues. Chromogenic images obtained from the immunostained tissue slides from each subject were analyzed using HALO software v3.4 (Indica Labs) for absolute quantitative IHC analysis. Results are expressed as mean ± SEM, and the non-parametric two-tailed Mann-Whitney test was used to determine the differences in protein expression between COVID-19 and control postmortem human lung tissues for absolute quantitative IHC analysis (*, *p* < 0.05; **, *p* < 0.01).

Although no significant difference was observed in protein expression for the investigated inflammatory markers and regulatory proteins (Figure 5), the efflux transporters P-gp and MRP1 reached a significant difference in protein expression between the COVID-19 and control groups (Figure 6B). No significant differences in protein expression for the investigated DMEs was observed (Figures 5, 6).

## Discussion

In this study, we investigated the effect of SARS-CoV-2 infection in the dysregulation of 25 clinically relevant DMETs at the mRNA and protein levels in Vero E6 cells and postmortem human lung tissues obtained from COVID-19 patients, respectively. In postmortem human lung tissues, we further assessed biomarker localization and the role of SARS-CoV-2-associated inflammatory response and xenosensing regulatory proteins in modulating the dysregulation of DMETs. Our study led to three major outcomes:

### SARS-CoV-2-infected Vero E6 cells demonstrated dysregulation of the metabolic enzyme involved in the disposition of commonly prescribed COVID-19 drugs

With the SARS-CoV-2-associated inflammatory response in Vero E6 cells, we observed that at the mRNA level, *CYP3A4* was upregulated while *UGT1A1* was downregulated (Figure 2B). However, none of the investigated transporters were significantly dysregulated (Figure 2C and Supplementary Figure S3B). Several disease-drug interactions associated with SARS-CoV-2 infection have been suggested (Kumar and Trivedi, 2021). For example, CYP3A4 involved in the metabolism of dexamethasone, a corticosteroid drug often administered to hospitalized COVID-19 patients, is known to be dysregulated by inflammation (Kumar and Trivedi, 2021; Dunvald et al., 2022). Also, nirmatrelvir, an antiviral drug used to treat SARS-CoV-2 infection is metabolized by CYP3A4 and is currently coadministered with ritonavir (an inhibitor of CYP3A4) to improve its bioavailability (Nwabufo and Bendayan, 2022). Therefore, it is important to further investigate how SARS-CoV-2-associated inflammatory response will affect the PK profile of nirmatrelvir-ritonavir combination therapy for the treatment of COVID-19. Furthermore, human clinical data suggests that remdesivir is extensively metabolized by CYP2C8, CYP2D6, and CYP3A4 (Yang, 2020) and since studies have shown that these enzymes are dysregulated by inflammation (Dunvald et al., 2022), the PK profile of remdesivir and dexamethasone could be altered by the inflammatory state observed in COVID-19. This may partly explain the variable clinical outcomes observed in several clinical studies that investigated the safety and efficacy of remdesivir (Cascella et al., 2022). In general, remdesivir was primarily approved by the US FDA for administration to COVID-19 patients requiring hospitalization, and proper dosing regimen and treatment course need to be further investigated to achieve the full benefits of remdesivir for patients at different stages of COVID-19. Additionally, caution needs to be taken when administering drugs to COVID-19 patients especially the patient population with comorbidities to mitigate clinically relevant disease–drug interactions. For example, UGT1A1 is highly polymorphic and can impact irinotecan (a prodrug used for small cell lung cancer chemotherapy) metabolite related-toxicity (Bandyopadhyay et al., 2021). Given that *UGT1A1* mRNA expression was significantly downregulated in SARS-CoV-2-infected Vero E6 cells and patients with lung cancer have a greater than 7-fold higher risk of SARS-CoV-2 infection (Rolfo et al., 2022), further investigation is required to determine the effect of prescribing UGT1A1 candidate drugs to COVID-19 patients, especially the UGT1A1 poor metabolizers which accounts for about 10% of North Americans (Dean, 2018).

Dunvald and coworkers recently summarized studies reporting altered mRNA expression of clinically relevant DMETs including *CYP1A2*, *CYP2B6*, *CYP2C8*, *CYP2C9*, *CYP2C19*, *CYP2D6*, *CYP3A4*, *PGP*, *BCRP*, and *MRP2* by proinflammatory cytokines such as IL-6, IL-1β, TNF-α, and IFN-γ in a dose-dependent manner in primary cultures of human hepatocytes (Dunvald et al., 2022). For example, the mRNA expression of the hepatic efflux transporters P-gp, MRP2, and BCRP is typically low, with a maximum downregulation of 2-fold, in response to IL-6, IL-1β, TNF-α, or IFN-γ at doses of 1–100 ng/mL (Ramsden et al., 2015; Moreau et al., 2017; Ning et al., 2017; Dunvald et al., 2022). However, the concentrations of the investigated inflammatory markers in SARS-CoV-2 infected Vero E6 cells are probably more physiologically relevant compared to the artificial stimulation of inflammatory response by the administration of toxins such as lipopolysaccharides, or direct administration of proinflammatory cytokines such as IL-6 and IL-1β—both of which may result in supraphysiological responses. Furthermore, species (human and monkey) and organ (kidney and liver) differences in DMETs expression, may be responsible for the lack of dysregulation of the above-mentioned DMETs. For example, a previous study found that *CYP2C18* mRNA expression is unaffected by cytokine administration due to limited hepatic expression (Aitken and Morgan, 2007).

### DMETs are expressed in several human pulmonary cells affected by SARS-CoV-2 infection

We localized for the first time, the cellular expression of uptake transporters—ENT1 and ENT2 in human lung tissues by IHC. We found that both ENT1 and ENT2 were primarily localized in inflammatory cells of lung tissues from COVID-19 patients (Figure 4; Table 2), making them potentially liable to SARS-CoV-2-mediated increases in expression. Interestingly, ENT1 and ENT2 may be prone to downregulation with SARS-CoV-2 infection due to the presence of acute lung injury and hypoxia in some COVID-19 patients (Johnson, 2022). Indeed, previous studies reported a significant downregulation of ENT1 and ENT2 expression in lung epithelial and endothelial cells in both acute lung injury (Morote-Garcia et al., 2013) and hypoxia (Eltzschig et al., 2005). More so, increased extracellular adenosine levels due to acute lung injuries (Eckle et al., 2009) suggest a potential competitive inhibition of ENTs-mediated transport processes. This is clinically important because ENT1 and ENT2 are involved in the uptake of the COVID-19 drugs - remdesivir and molnupiravir, making this uptake pathway potentially liable to dysregulation with SARS-CoV-2 infection and may partly explain the limited and variable clinical efficacy observed for remdesivir (Johnson, 2022).

To the best of our knowledge, our study is the first to reveal the cellular localization of CYP1A2, CYP2C8, CYP2C9, and CYP2D6 by IHC in human lung tissues. Previous studies investigating the expression of DMEs in the human respiratory system, have mostly assessed expression at the mRNA level, used non-intact lung samples such as microsomes and bronchial specimens, or failed to uncover cellular localization (Hukkanen et al., 2002). The human lung tissue is highly heterogeneous, making microsomes, bronchial, and other non-intact lung specimens an inaccurate description of DMETs expression in different pulmonary cell types. For example, the mRNA expression of CYP2C8 was previously found in both bronchial and peripheral lung tissue samples (Macé et al., 1998), but its protein expression and cellular localization were not reported. An IHC study identified CYP2B6 in human Clara cells (Mori et al., 1996) whereas CYP2C19 and CYP3A4 proteins were detected in serous cells of bronchial glands (Yokose et al., 1999) but expression in other pulmonary cell types was not reported. Our study uncovered the pulmonary cellular localization of CYP2B6, CYP2C8, CYP2C19, and CYP3A4 (Figure 4; Table 2).

Efflux transporters such as P-gp and BCRP have previously been identified in human lung tissues (Cordon-Cardo et al., 1990; Van der Valk et al., 1990; Scheffer et al., 2002; Fetsch et al., 2006). In contrast to an earlier study that found BCRP expression only in bronchial epithelial cells and capillaries (Scheffer et al., 2002), later work demonstrated staining of alveolar pneumocytes and negligible staining of the bronchial epithelial cells (Fetsch et al., 2006). These findings are not in agreement with the results of this study. In our study, BCRP was primarily localized in the cytoplasm of endothelial cells in both COVID-19 and control tissues (Figure 4; Table 2). In previous reports, P-gp localization was shown on the luminal surface of bronchial and bronchiolar epithelial cells (Cordon-Cardo et al., 1990; Van der Valk et al., 1990). P-gp was stained in alveolar macrophages whereas staining of alveolar epithelial cells was dependent on the antibody employed (Van der Valk et al., 1990). On the contrary, our findings showed that P-gp is robustly localized in the alveolar epithelial cells (Figure 4; Table 2) of both COVID-19 and control tissues. MRP1 was initially found in the apical membrane of the cytoplasm of bronchial epithelial cells (Flens et al., 1996); however, two later studies confirmed MRP1 localization in the basolateral membrane (Bréchot et al., 1998; Scheffer et al., 2002) which is consistent with its localization in other tissues (Gustavsson et al., 2016). In our study, MRP1 expression in the bronchial epithelium is cytoplasmic (Figure 4; Table 2). Moreover, MRP1 was predominantly localized in the cytoplasm and nucleus of alveolar epithelial cells of both COVID-19 and control postmortem human lung tissues (Figure 4; Table 2). MRP2 positivity was previously found in the apical membrane of the bronchial and bronchiolar epithelial layers (Sandusky et al., 2002; Scheffer et al., 2002). We observed a robust localization in the alveolar epithelial cells of both COVID-19 and control tissues (Table 2). We also found MRP2 expression in bronchial epithelial cells in a higher number for controls (3/5) compared to the COVID-19 (1/10) human lung tissues; however, it was more circumferential not apical membranous compartmentalization (Figure 4; Table 2). Our study demonstrated that circumferential membrane compartmentalization in alveolar epithelial and endothelial cells was the major expression site for MRP2 (Figure 4; Table 2). This circumferential compartmentalization is indicative of potential bidirectional efflux transport processes mediated by MRP2 in human lung tissues compared to the anticipated unidirectional efflux transport.

Typically DMETs are localized in the cytoplasm and cell membrane, however, our study found that some DMETs were localized in atypical cellular compartments (Figure 4; Table 2). For example, our study detected MRP1 in the cytoplasm and nucleus. Although transporters are localized at the cell membrane, they are also present in cytoplasmic organelles such as the Golgi apparatus, rough endoplasmic reticulum, nuclear envelope, and mitochondria. For instance, a previous IHC study found MRP2 expression in both cytoplasm and cell membrane of tumor cells (Yamasaki et al., 2011), and it is anticipated that in the cytoplasm, MRP2 may not function as an efflux pump (Evers et al., 1998). Therefore, further investigation is required to determine whether differences in pulmonary cellular compartmentalization affect the structure and function of the implicated DMETs.

### SARS-CoV-2 may dysregulate pulmonary DMETs through inflammatory response and tissue injuries

From the absolute quantitation, we observed significant differences in the expression of P-gp and MRP1 between the COVID-19 and control human lung tissues (Figure 6B). P-gp is involved in the transport of nirmatrelvir, remdesivir, and dexamethasone while MRP1 transports lopinavir and ritonavir (Nwabufo and Bendayan, 2022)—indicating a potential alteration in pulmonary drug PK/PD profile in COVID-19 patients. The lack of observable significant dysregulation in the protein expression of other DMETs in COVID-19 human lung tissues (Figures 5, 6) could be attributed to the potential variation in the stage of COVID-19 between cases, as well as comorbidities (Table 1), ongoing medications, and genetic polymorphisms in the expression of DMETs for both COVID-19 and control cases. These variables could not be further addressed due to the paucity of premortem clinical information. The small sample size, and other diseases present in the control samples may also have an impact on the expression of inflammatory markers, regulatory proteins, and DMETs, possibly resulting in a negligible dysregulation in their expression between COVID-19 and control tissues. Drug toxicity, cardiovascular diseases, and head trauma are the reported causes of death for the 5 control cases (Table 1), and these disorders can induce inflammatory response and dysregulate the expression of DMETs through regulatory proteins (Wu and Lin, 2019; Stanke-Labesque et al., 2020; Dunvald et al., 2022).

The congruency in the pulmonary cellular localization of SARS-CoV-2 infection, inflammatory markers, regulatory proteins, and DMETs, as well as the several COVID-19-related pulmonary pathologies of the COVID-19 cases suggest a potential dysregulation of pulmonary DMETs which may manifest in regulated clinical studies. We observed that SARS-CoV-2 infection and inflammatory response are distinctly localized to the cytoplasm of lymphocytes in COVID-19 compared to control human lung tissues (Figure 4; Table 2). The detection of SARS-CoV-2 viral protein in the control human lung tissues is indicative of the limited specificity of the antibody; however, clinical testing confirmed the presence of SARS-CoV-2 infection in the COVID-19 cases. Again, underlying diseases in the control cases are probably responsible for the observed inflammatory response. Interestingly, we observed a similar trend in the localization of xenosensing regulatory proteins; for example, the two master xenosensing regulatory proteins - PXR and CAR are both distinctly expressed in the cytoplasm of lymphocytes in COVID-19 human lung tissues but not in the control cases (Figure 4; Table 2). Also, we observed a similar trend in the cellular localization of some DMETs including CYP2C9, CYP2C19, CYP2D6, and MRP1 (Figure 4; Table 2). These observations suggest that SARS-CoV-2 infection could result in an inflammatory response that could activate xenosensing regulatory proteins, which could then dysregulate the expression of DMETs in lymphocytes. However, our study quantified global pulmonary protein expression for the investigated biomarkers to get a better representation of SARS-CoV-2-DMETs interactions. Moreover, it is practically challenging to quantify the investigated biomarkers in the lymphocytes of human lung tissues except when pulmonary lymphocyte isolates are used as specimens for the study.

Furthermore, we observed that the presence of inflammatory cells is a major driving force in the localization of DMETs between COVID-19 and control tissues (Figure 4; Table 2). In general, COVID-19 lung tissues had more inflammatory cells which also expressed DMETs compared to the control lung tissues. Notably, CYP3A4 and ENT2 were strongly localized in lymphocytes in COVID-19 compared to control human lung tissues (Figure 4; Table 2). This suggests a potential SARS-CoV-2-mediated increase in the expression of DMETs through the recruitment of inflammatory cells and may have implications for pulmonary drug PK/PD profiles. For example, CYP3A4 and ENT2 are involved in the disposition of remdesivir (Nwabufo and Bendayan, 2022) and may be susceptible to an altered pulmonary PK/PD profile in the context of SARS-CoV-2 infection. Additionally, the observed COVID-19-related pulmonary pathologies could also reduce the expression of DMETs. For example, DAD could alter the integrity of alveolar epithelial cells—which also house more than 90% of the investigated DMETs including CYP3A4 and P-gp. Our recent paper provides strategies for achieving optimal clinical efficacy and safety amidst SARS-CoV-2-associated inflammatory response (Nwabufo and Bendayan, 2022) and should be considered in the clinical decision-making process for COVID-19 drugs.

In conclusion, our study has shown for the first time that SARS-CoV-2 infection dysregulates clinically relevant DMEs—CYP3A4 and UGT1A1 at the mRNA level, and efflux transporters—P-gp and MRP1 at the protein level in Vero E6 cells and postmortem human lung tissues, respectively. We uncovered the human pulmonary localization of DMETs that are also involved in the disposition of COVID-19 drugs, and showed that inflammatory response is the driving force for the discrepancy in the localization of DMETs between COVID-19 and control human lung tissues. We observed that at the cellular level, DMETs could potentially be dysregulated by SARS-CoV-2-associated inflammatory responses and lung injuries. Further investigation of SARS-CoV-2-mediated dysregulation of human pulmonary DMETs and its potential implication in controlling the safety and efficacy of promising COVID-19 drugs as well as possible unexpected adverse drug reactions in COVID-19 patients on polypharmacy is needed. Further research is required to determine the spatial distribution and disposition of promising COVID-19 drugs at the cellular level in human lung tissues, and mass spectrometry imaging may offer an appealing analytical platform to further investigate this aspects (Nwabufo and Aigbogun, 2022).

## Data Availability

The original contributions presented in the study are included in the article/Supplementary Material, further inquiries can be directed to the corresponding author.
