# Supplementary material for: SARS-CoV-2 infection dysregulates the expression of clinically relevant drug metabolizing enzymes in Vero E6 cells and membrane transporters in human lung tissues
Source: Front Pharmacol. 2023 Apr 27;14:1124693. doi: 10.3389/fphar.2023.1124693 (PMC10172598; doi:10.3389/fphar.2023.1124693)
Supplement: Supplementary file 1 [file Table1.docx]

**Supplementary Material**

**SARS-CoV-2 Infection Dysregulates the Expression of Clinically Relevant Drug Metabolizing Enzymes in Vero E6 Cells and Membrane Transporters in Human Lung Tissues**

Chukwunonso K. Nwabufo^1, 2^*****, Md. Tozammel Hoque^1^, Lily Yip^3^, Maliha Khara ^4, 5^, Samira Mubareka^3, 5^, Michael S Pollanen^4, 5^, and Reina Bendayan^1^

^1^Department of Pharmaceutical Sciences, Leslie Dan Faculty of Pharmacy, University of Toronto, Toronto, ON, Canada.

^2^OneDrug, Toronto, ON, Canada.

^3^Sunnybrook Research Institute, Toronto, ON, Canada.

^4^Ontario Forensic Pathology Service, Toronto, ON, Canada.

^5^Department of Laboratory Medicine and Pathobiology, University of Toronto, Toronto, ON, Canada.

***Corresponding Author**

Chukwunonso Nwabufo:<mailto:> [Chukwunonso.nwabufo@usask.ca](mailto:Chukwunonso.nwabufo@usask.ca); Chukwunonso.nwabufo@mail.utoronto.ca

Department of Pharmaceutical Sciences

Leslie Dan Faculty of Pharmacy, University of Toronto

144 College Street, Toronto, ON, M5S 3M2

**Supplementary Information**

**
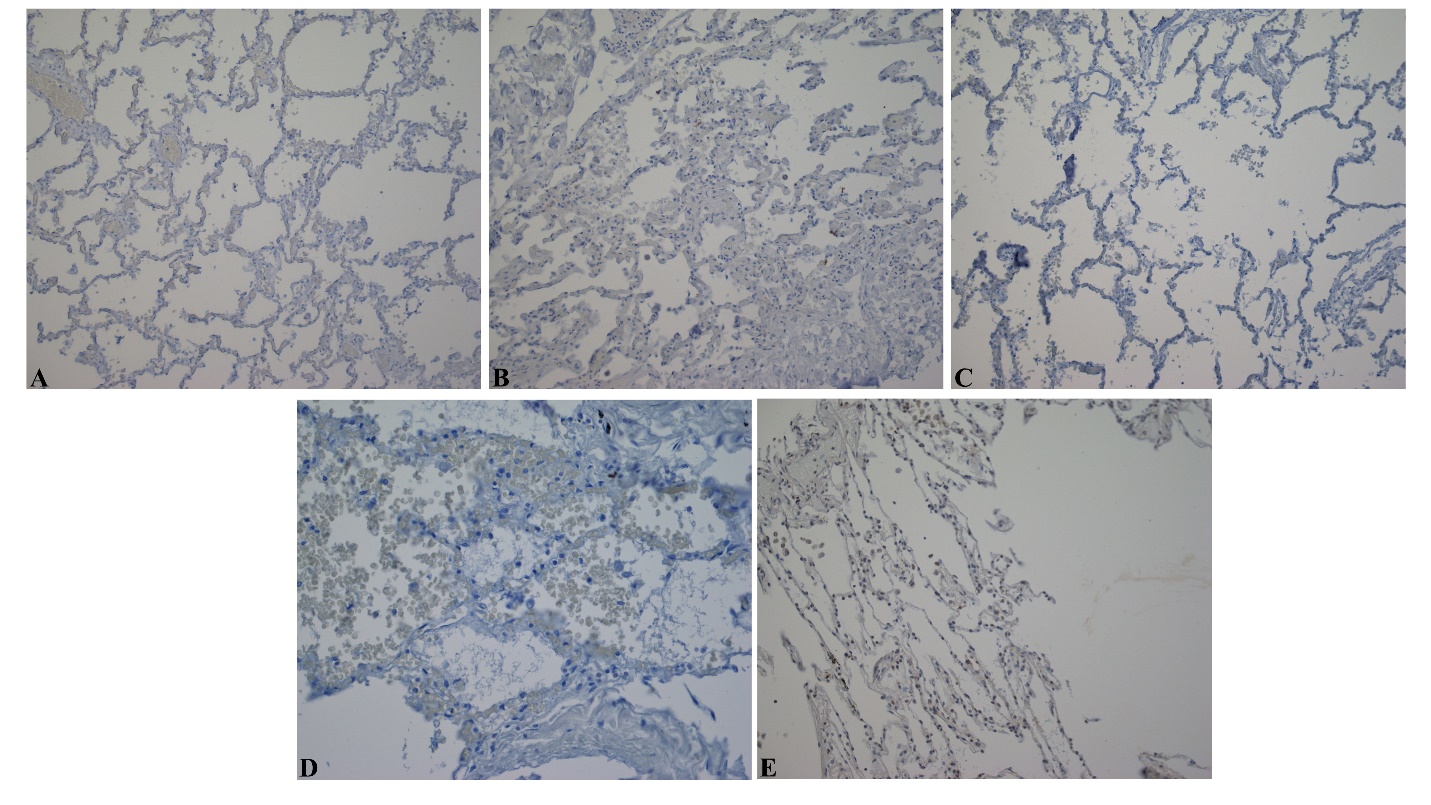
**

**Supplementary Figure S1.** Micrographs showing reagent negative control human lung tissues (40x magnification with an Olympus BX43 microscope) that indicated no positive staining in the absence of primary antibody. Some reagent negative control slides are a representation of other investigated biomarkers that had the same pre-treatment reagents as shown in Table S5, including: **A** (CYP1A2, CYP2C19, ENT2, MRP1, IL-1β, IL-6, CAR, and SARS-CoV2 spike protein); **B** (CYP2B6, CYP2C8, CYP2C9, CYP2D6, CYP3A4, MRP2, P-gp, NF-kβ); **C** (BCRP, pSTAT3, PXR); **D** (ENT1); **E** (SARS-CoV-2 nucleocapsid protein)

**
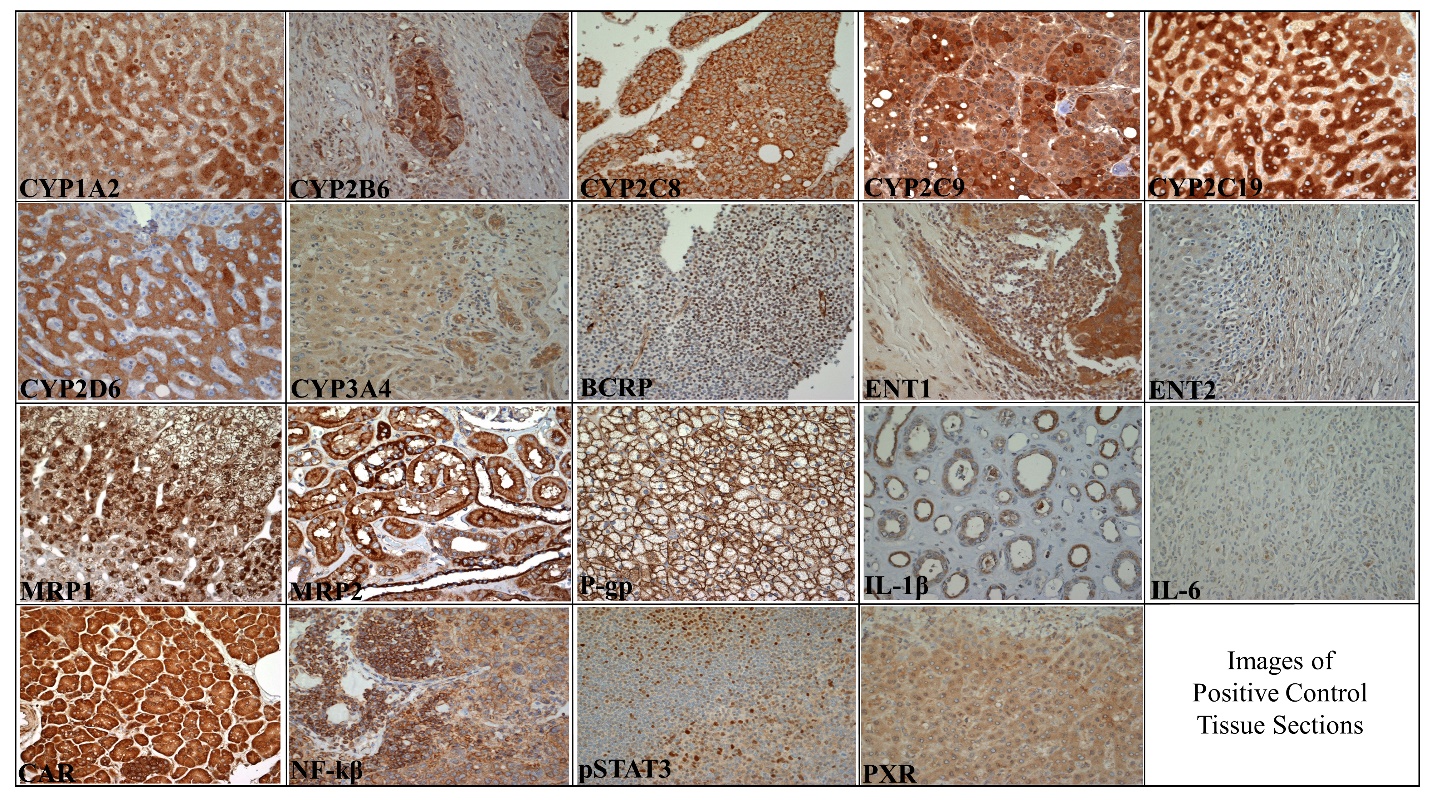
Supplementary Figure S2.** Micrographs showing positive staining in brown for the investigated biomarkers (40x magnification with an Olympus BX43 microscope). The positive control tissues include normal liver (CYP1A2, CYP2C19, CYP2D6, CYP3A4, and PXR); liver tumor (CYP2C8, and CYP2C9); colon tumor (CYP2B6, NF-kβ); bladder (ENT2 and IL-6); adrenal (P-gp and MRP1); breast tumor (ENT1); tonsil (BCRP and pSTAT3); kidney (MRP2 and IL-1β); and pancreas (CAR)

**
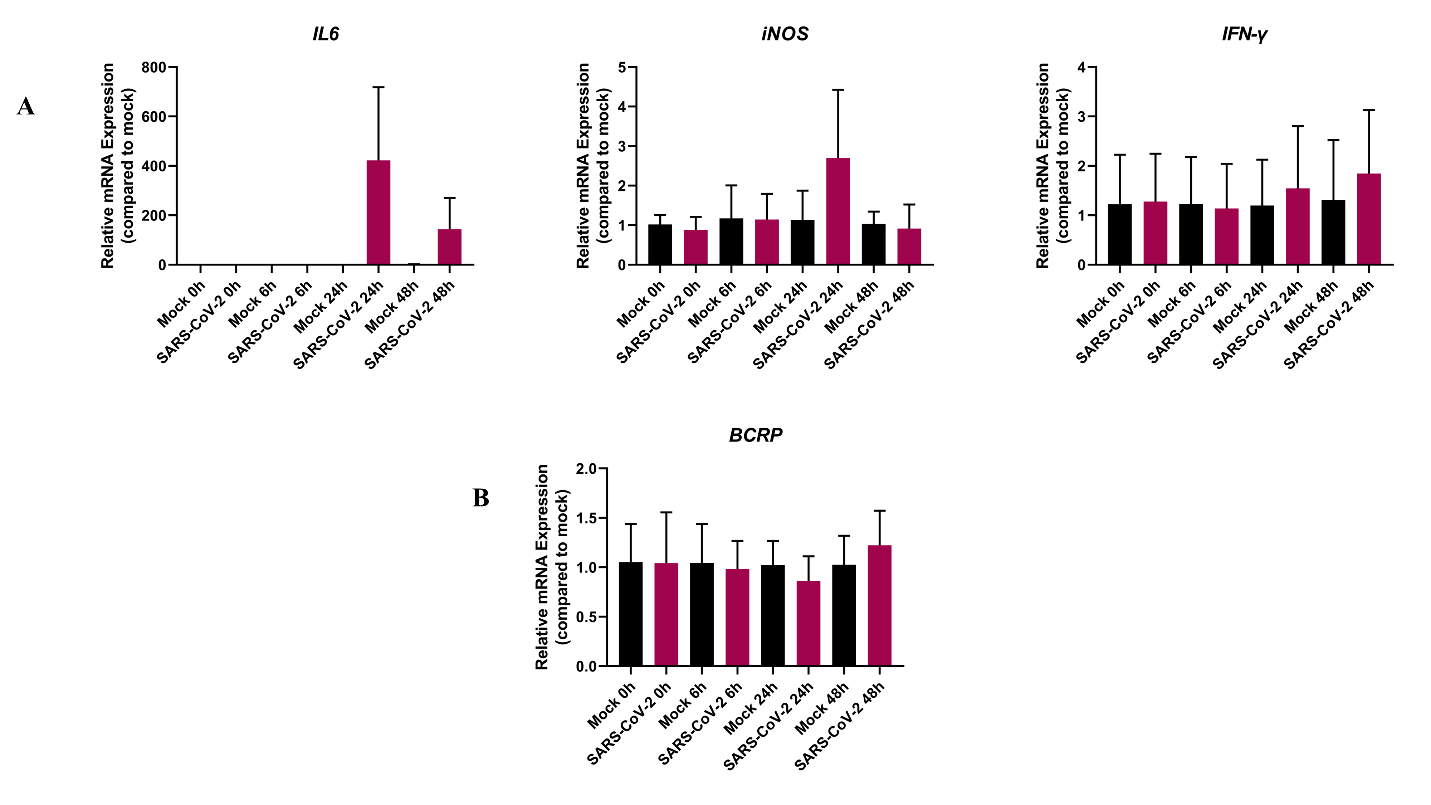
**

**Supplementary Figure S3.** Effect of SARS-CoV-2 infection on the mRNA expression of selected (**A**) inflammatory markers and (**B**) membrane-associated drug transporter in Vero E6 cells. Relative mRNA expression was determined using qRT-PCR with normalization to the housekeeping gene and the mock. Results are expressed as mean ± SD from 3 independent experiments, and unpaired t-test was used to determine significant differences.

**Supplementary Table S1.** List of Observed Single Nucleotide Variations and their Frequencies

| S357_P2_LY | | | | | |
| --- | --- | --- | --- | --- | --- |
| Position | Mutation | Frequency | Annotation | Gene | Description |
| 241 | C→T | 100% | intergenic (–/-25) | – / → orf1ab | –/orf1ab polyprotein |
| 686 | Δ9 bp | 100% | coding (421-429/13203 nt) | orf1ab → | orf1ab polyprotein |
| 1059 | C→T | 100% | T265I (ACC→ATC) | orf1ab → | orf1ab polyprotein |
| 3037 | C→T | 100% | F924F (TTC→TTT) | orf1ab → | orf1ab polyprotein |
| 8092 | C→T | 100% | L2609L (CTC→CTT) | orf1ab → | orf1ab polyprotein |
| 14408 | C→T | 100% | P314L (CCT→CTT) | orf1ab → | orf1ab polyprotein |
| 23403 | A→G | 100% | D614G (GAT→GGT) | S → | surface glycoprotein |
| 23525 | C→T | 10% | H655Y (CAT→TAT) | S → | surface glycoprotein |
| 23598 | Δ21 bp | 7% | coding (2036-2056/3822 nt) | S → | surface glycoprotein |
| 23628 | Δ9 bp | 10% | coding (2066-2074/3822 nt) | S → | surface glycoprotein |
| 25324 | C→A | 17% | C1254* (TGC→TGA) | S → | surface glycoprotein |
| 25325 | A→T | 7% | K1255* (AAA→TAA) | S → | surface glycoprotein |
| 25563 | G→T | 100% | Q57H (CAG→CAT) | ORF3a → | ORF3a protein |
| 27059 | C→T | 13% | Y179Y (TAC→TAT) | M → | membrane glycoprotein |
| 29234 | G→A | 100% | G321S (GGC→AGC) | N → | nucleocapsid phosphoprotein |

**Supplementary Table S2.** Panel of Biomarkers Investigated in Mock and SARS-CoV-2 infected Vero E6 Cells

| **Drug Metabolizing Enzymes** | **Membrane-associated Drug Transporters** | **Inflammatory Markers** |
| --- | --- | --- |
| CYP1A2 | P-gp | IL-6 |
| CYP2B6 | MRP1 | IL-1β |
| CYP2C8 | MRP2 | IFN- γ |
| CYP2C9 | BCRP | TNF-α |
| CYP2D6 | MRP4 | iNOS |
| CYP3A4 | — | CCL2 |
| UGT1A1 | — | CXCL10 |
| — | — | CRP |
| — | — | IL-10 |

**Supplementary Table S3.** Primers used for qRT-PCR Analyses

| **Gene Symbol** | **Protein** | **Assay identification Number** |
| --- | --- | --- |
| **Inflammatory markers** | | |
| *IL6* | IL-6 | Rh02621719_u1 |
| *IL1β* | IL-1β | Rh02621711_m1 |
| *IFN- γ* | IFN- γ | Rh02621721_m1 |
| *TNF* | TNF-α | Rh02621718-s1 |
| *NOS* | iNOS | Rh01075507_m1 |
| *CCL2* | CCL2 | Rh02621753_m1 |
| *CXCL10* | CXCL10 | Rh02788358-m1 |
| *CRP* | CRP | Rh02902752_m1 |
| *IL10* | IL-10 | Rh00961619_m1 |
| **Drug Metabolizing Enzymes** | | |
| *CYP1A2* | CYP1A2 | Mf04946246_m1 |
| *CYP2B6* | CYP2B6 | Rh03043066_m1 |
| *CYP2C8* | CYP2C8 | Rh02790965_uH |
| *CYP2C9* | CYP2C9 | Rh02789928-m1 |
| *CYP2D6* | CYP2D6 | Rh02826836_m1 |
| *CYP3A4* | CYP3A4 | Rh02872540_m1 |
| *UGT1A1* | UGT1A1 | Rh02828741_m1 |
| **Membrane-associated drug transporters** | | |
| *ABCB1* | P-gp | Rh01070639_m1 |
| *ABCC1* | MRP1 | Rh01561504_m1 |
| *ABCC2* | MRP2 | Rh00960504_m1 |
| *ABCG2* | BCRP | Rh01053786_m1 |
| *ABCC4* | MRP4 | Rh00988713_m1 |
| **Housekeeping genes** | | |
| *PPIB* | Peptidylprolyl isomerase B | Rh01018503_m1 |
| *GAPDH* | Glyceraldehyde-3-phosphate dehydrogenase | Rh02621745_g1 |

| **Drug Metabolizing Enzymes** | **Membrane-associated Drug Transporters** | **Regulatory Markers** | **Inflammatory Marker** |
| --- | --- | --- | --- |
| CYP1A2 | P-gp | PXR | IL-6 |
| CYP2B6 | MRP1 | CAR | IL-1β |
| CYP2C8 | MRP2 | NF-kβ | — |
| CYP2C9 | BCRP | pSTAT3 | — |
| CYP2C19 | ENT1 | — | — |
| CYP2D6 | ENT2 | — | — |
| CYP3A4 | — | — | — |
|  |  |  |  |

**Supplementary Table S4.** Panel of Biomarkers Investigated in COVID-19 and Control Postmortem Human Lung Tissues

**Supplementary Table S5.** Antibodies, Dilutions, and Pre-treatment Conditions used for Chromogenic Immunohistochemistry Analyses

| **Antibody Name** | **Antibody Type (Clonality)** | **Supplier (Catalog #)** | **Dilution** | **Pretreatment** | **Incubation** |
| --- | --- | --- | --- | --- | --- |
| Anti-P-gp | Mouse Monoclonal (D-11) | Santa Cruz Biotechnology (sc-55510) | 1:300 | HIER Tris-EDTA, LT | Overnight, RT |
| Anti-MRP1 | Rat Monoclonal (MRPr1) | Kamiya Biomedical Company (MC-201) | 1:200 | HIER Citrate, LT | Overnight, RT |
| Anti-MRP2 | Mouse Monoclonal (M2III-6) | Kamiya Biomedical Company (MC-206) | 1:300 | HIER Tris-EDTA, LT | Overnight, RT |
| Anti-BCRP | Rat Monoclonal (BXP-53) | Enzo (ALX-801-036-C050) | 1:500 | HIER Tris-EDTA | Overnight, RT |
| Anti-CYP1A2 | Rabbit Polyclonal | Proteintech (19936-1-AP) | 1:500 | HIER Citrate, LT | Overnight, RT |
| Anti-CYP2B6 | Rabbit Polyclonal | Abcam (ab198870) | 1:200 | HIER Tris-EDTA , LT | 1 h, RT |
| Anti-CYP2C8 | Rabbit Polyclonal | Proteintech (16546-1-AP) | 1:1000 | HIER Tris-EDTA , LT | 1 h, RT |
| Anti-CYP2C9 | Rabbit Polyclonal | Abcepta (AP7881c) | 1:300 | HIER Tris-EDTA , LT | 1 h, RT |
| Anti-CYP3A4 | Rabbit Polyclonal | Abcam (ab135813) | 1:250 | HIER Tris-EDTA , LT | Overnight, RT |
| Anti-CYP2D6 | Rabbit Monoclonal (EPR17868) | Abcam (ab185625) | 1:500 | HIER Tris-EDTA , LT | 1 h, RT |
| Anti-CYP2C19 | Rabbit Polyclonal | Invitrogen (PA5-114368) | 1:500 | HIER Citrate, LT | Overnight, RT |
| Anti-NF-Kb p65 | Rabbit Monoclonal (E379) | Abcam (ab32536) | 1:2000 | HIER Tris-EDTA , LT | 1 h, RT |
| Anti-STAT3 (phospho Y705) | Rabbit Monoclonal (EP2147Y) | Abcam (ab76315) | 1:100 | HIER Tris-EDTA | Overnight, RT |
| Anti-CAR | Rabbit Polyclonal | Abcam (ab186869) | 1:1000 | HIER Citrate, LT | 1 h, RT |
| Anti-PXR (G-11) | Mouse Monoclonal IgG | Santa Cruz Biotechnology (sc-48403) | 1:50 | HIER Tris-EDTA | Overnight, RT |
| Anti-SARS-CoV-2 Nucleocapsid protein | Rabbit Polyclonal | Novus (NB100-56576SS) | 1:500 | Citrate, Pepsin, LT | Overnight, RT |
| Anti-SARS-CoV-2 Spike protein | Rabbit Polyclonal | Abcam (ab272504) | 1:5000 | HIER Citrate, LT | Overnight, RT |
| Anti-ENT1 | Rabbit Polyclonal | Proteintech (11337-1-AP) | 1:200 | NPTM | Overnight, RT |
| Anti-ENT2 | Rabbit Polyclonal | Invitrogen (PA5-38006) | 1:500 | HIER Citrate, LT | 1 h, RT |
| Anti-IL-6 | Rabbit Polyclonal | Abcam (ab216492) | 1:300 | HIER Citrate, LT | 1 h, RT |
| Anti-IL-1β | Rabbit Polyclonal | Abcam (ab2105) | 1:500 | HIER Citrate, LT | 1 h, RT |

HIER, heat induced epitope retrieval; LT, low temperature antigen retrieval; RT, room temperature; NPTM, no pretreatment
